# Site-Mutation of Hydrophobic Core Residues Synchronically Poise Super Interleukin 2 for Signaling: Identifying Distant Structural Effects through Affordable Computations

**DOI:** 10.3390/ijms19030916

**Published:** 2018-03-20

**Authors:** Longcan Mei, Yanping Zhou, Lizhe Zhu, Changlin Liu, Zhuo Wu, Fangkui Wang, Gefei Hao, Di Yu, Hong Yuan, Yanfang Cui

**Affiliations:** 1 Key Laboratory of Pesticide and Chemical Biology, Ministry of Education, Central China Normal University, Wuhan 430079, China; mlc2015@mails.ccnu.edu.cn (L.M.); hgzyp@mail.ccnu.edu.cn (Y.Z.); liuchl@mail.ccnu.edu.cn (C.L.); wuzhuo@mails.ccnu.edu.cn (Z.W.); gfhao@mail.ccnu.edu.cn (G.H.); 2 Department of Chemistry, Hong Kong University of Science and Technology, Clear Water Bay, Hong Kong 999077, China; lizhezhu11@gmail.com; 3 State Key Lab of Agricultural Microbiology, Huazhong Agricultural University, Wuhan 430070, China; wangfangkui@foxmail.com; 4 Department of Immunology and Infectious Disease, John Curtin School of Medical Research, The Australian National University, Acton 2601, Australia; di.yu@anu.edu.au; 5 Department of Biochemistry and Molecular Biology, Biomedicine Discovery Institute, Monash University, Clayton 3800, Australia

**Keywords:** ensemble allosteric model, dynamic network, cross-correlation, dynamic network pathways

## Abstract

A superkine variant of interleukin-2 with six site mutations away from the binding interface developed from the yeast display technique has been previously characterized as undergoing a distal structure alteration which is responsible for its super-potency and provides an elegant case study with which to get insight about how to utilize allosteric effect to achieve desirable protein functions. By examining the dynamic network and the allosteric pathways related to those mutated residues using various computational approaches, we found that nanosecond time scale all-atom molecular dynamics simulations can identify the dynamic network as efficient as an ensemble algorithm. The differentiated pathways for the six core residues form a dynamic network that outlines the area of structure alteration. The results offer potentials of using affordable computing power to predict allosteric structure of mutants in knowledge-based mutagenesis.

## 1. Introduction

Mutation of one or more residues into other amino acids through computational or experimental methods is often needed to attain a desired function in protein engineering for the purpose of either academia or industry [1]. When structure-related information such as binding interface or active site is available for the protein under study, a rational knowledge-based mutagenesis strategy is often employed by substituting residues on the binding interface or active site, in part due to the difficulty to predict the structural and, thus, functional effect from a distal residue mutation. However, determined structures of protein and their variants have proven that structural alterations that result from site-directed mutations quite often take place not only in local areas but also in distant regions through ubiquitous allosteric effects. The advancing direct evolution strategy of protein engineering has provided an increasing number of functional mutants more complex than that mutated on the binding surface or active site, which includes valuable subjects that could be analyzed to reveal the long distance effect of mutations [2,3,4].

The immunostimulatory cytokine Interleukin-2 (IL-2) has shown potency for autoimmune disease treatment [5,6,7,8] as well as adverse effects [9,10,11], which deserve more investment to develop its therapeutic potential. The type I cytokine exists as a bundle of four α-helices, in which the inner center of the helical bundle forms a hydrophobic core region. A mutant D10 developed from the yeast surface display technique was termed as a “superkine” since it presents increased activity by about ten times relative to its wild type IL-2 [12]. Crystal structure of the derivative of substituting six core residues away from the binding sites (helices A and C) to IL-2Rβ revealed that the mutations caused such allosteric structure alteration that the mutant structure became closer to the IL-2Rβ bound state structure of IL-2 and enhanced its binding affinity for IL-2Rβ [12]. However, it has not been interrogated regarding how the mutated residues located in the core region away from the binding interface influence the conformation and, thus, increase binding affinity and activity.

Structural change caused by distant residue mutation due to conformational allosteric effect could be the outcome of sparse energetic networks or dynamical coupling between amino acids. This notion was previously noticed [13] and carefully explored [14,15]. Ever since then, protein studies have been reporting a surge in experimental determination of dynamic network or amino acid network [16,17,18]. Now, it has been increasingly accepted that dynamic coupling between amino acids at the global level is a critical deterministic factor of protein functions [19,20], which motivated us to investigate how the hydrophobic core residues in IL-2 superkine affect its signaling potency by examining its molecular dynamics network. Of note, Lewis E. Kay’s lab using Nuclear Magnetic Resonance (NMR) relaxation experiment demonstrated the coupled network in cyclophilin A (CypA), a protein of 164 amino acids consisting of β-sheets. They proved that intrinsic plasticity of the protein is a necessary key characteristic of its catalysis function, and motion of atoms accounting for the intrinsic plasticity is localized not only at the active site but also along a wider dynamic network [21]. The “dynamic network” was well determined and designated. Dynamically coupled residues in a protein formed the dynamic network and the structural alteration regions from site mutations that coincide with the underlying range of the dynamic network. Further establishment of this notion has the potential of predicting the range of conformational change from site mutations throughout the entire protein molecule.

Computational programs could provide information of the protein dynamics networks in compensatory to experimental measurement [22,23,24,25,26,27,28] that is solid but not often feasible due to limitation on cost or sensitivity. Atomic-level molecular dynamics (MD) simulations based on empirical potentials give a detailed view of the dynamic evolution of the molecule. MD describes the dynamically coupled portions of a protein as “cross-correlation” and the cross-correlated portions in a protein could be either correlated (motions have the same phase) or anti-correlated motions (motions have opposite phase). Both kinds are responsible for mediating the enzyme catalysis [29]. The correlated residues can be graphed into amino acid networks where the residues are treated as nodes and the correlation edges according to graph theory [30]. The protein motions that are globally wired and resulting in conformational changes take place on timescales approximately of 200 μs to 10 ms where the rate constants are in the range of 100–5000 s^−1^, which is detected by NMR relaxation dispersion experiments for CypA [21]. The structural alteration caused by a mutant on this timescale can in principle be simulated via brute-force all-atom MD simulations [31], for example, microsecond level MD simulation revealed conformational difference of the active site of enzyme LovD and its variants caused by remote mutations [4]. Nonetheless, this is often so restrained by limited available computing facilities in a large number of low intensively computational bases. Therefore, using affordable computing power to predict the long-range mutational effects warrants efforts.

Unlike MD simulations map coupling amino acids through temporally recording the trajectory of the atoms in the amino acid networks, the ensemble allosteric model (EAM) highlights the statistical nature of the interactions of the coupled amino acids based on the belief that the coupling process of the wiring amino acids involves multiple conformational states. Therefore, the coupling could be manifested from changes of the conformational distribution or thermal fluctuations. This model detects energy propagation in terms of “cooperative network” by monitoring the coupling of the responses for each residue pair. When a residue at a site is susceptible to a perturbation at another site, the residue pair is denoted as cooperative [32,33]. The ensemble model provides a potential framework for interpreting long-time scale MD simulations of allosteric proteins. Therefore, the derived dynamic networks have the potential to reflect the result from a long-time scale MD simulation.

It is conceivable that cross-correlations in proteins could be derived from a shorter trajectory of movements than that needed for conformational characterization especially when considering that conformational alteration is the result of energetic propagation. To date, most documented cross-correlation maps in proteins or their complexes have been obtained through nanosecond timescale MD simulations [2,3,4] although the timescale issue has not been carefully examined. Here, the hypothesis that nanosecond timescale of conventional MD simulation could efficiently reveal the conformational allosteric effects that occur at a timescale of micro-seconds to milli-seconds was tested by taking advantage of the available experimental dynamic network in CypA, which was once proven to be overlaid with the structural changes area (the areas that underwent chemical-shift changes). We derived the dynamic network from 100 ns MD trajectory and compared it with the experiment which was proven to be at the level of microsecond and found high consistency between the two, which suggests that nanosecond MD trajectory for a protein of CypA size is able to map the dynamics network and the regions of this network predict the regions that are most likely to undergo structural changes by site mutation.

Nanosecond MD simulation was subsequently used to detect the dynamic networks in wild type IL-2 and D10. Six differentiated dynamic pathways were figured out, the sum of which formed an overall dynamic network located within or very close to the residues undergoing remarkable structural changes. Another single chain protein, IL-1β, was used to further test the notion and similar results were obtained. The EAM method was employed to detect the dynamic networks as a reference to the result of long-time scale MD simulations. In all, the dynamic pathways forming the dynamic network through which mutations in IL-2 superkine organize the residues to poise closer to its bound state were deciphered in the present study. The dynamic network could be computed economically, which holds potential to predict an effective range of structural alterations caused by site mutations and could then be integrated in the knowledge-based rational design of proteins.

## 2. Results

### 2.1. The Correlation Pattern in CypA Detected by MD Simulation Coincides with the Experimentally Detected Dynamic Networks

**Figure 1:**
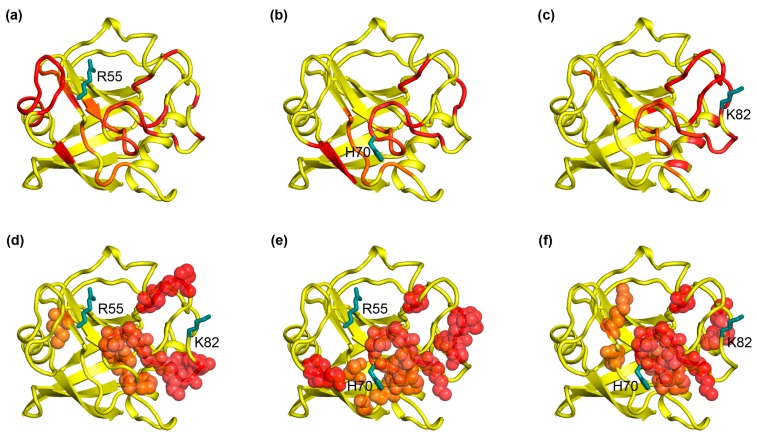
The dynamic network in CypA detected by NMR experiments. The common dynamic network has been reported in the literature. The residues with chemical shift changes in single-mutation R55A (**a**), H70A (**b**) and K82A (**c**) are colored in red. The residues with chemical shift changes in double-mutation R55A and K82A (**d**), R55A and H70 (**e**) and H70A and K82A (**f**) are colored in red and represented as spheres.

**Figure 2:**
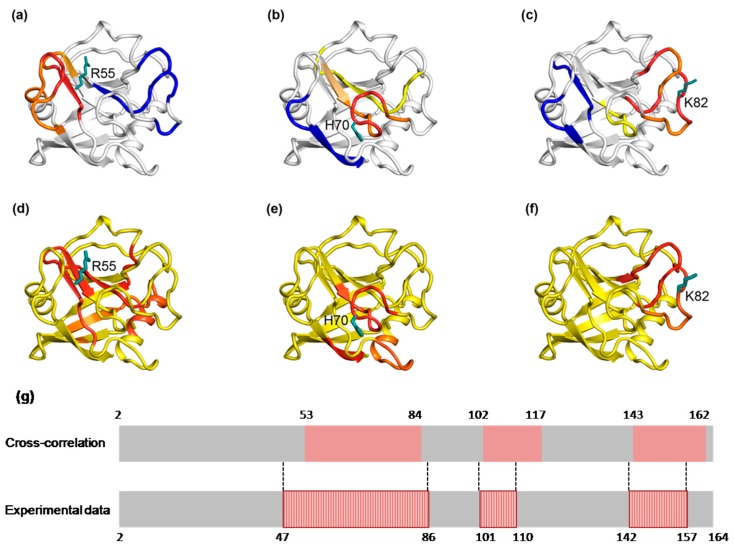
The dynamic network in CypA deduced by cross-correlation analysis and COREX/BEST algorithm. (**a**–**c**) The dynamic network deduced by cross-correlation analysis. The dominant correlated pattern of the residues in CypA is shown relative to residues R55 (**a**), H70 (**b**) and K82 (**c**), respectively. The residues color coded red, orange and yellow are correlated, while these residues are anticorrelated with those color-coded blue. (**d**–**f**) The dynamic network deduced by COREX/BEST algorithm. The energetic coupling regions in the network for R55A (**d**), H70A (**e**) and K82A (**f**) are colored in red. The effects of single Ala substitution on all other residues are quantified by calculating the change in free energy. For clarity the coupling regions only consist of the residues with the free energy change greater than 0.2 kcal/mol. (**g**) Comparison of the dynamic network calculated by cross-correlation analysis and NMR experiments. Along the protein sequence, red regions indicate residues within the dynamic network. Boxes with vertical bars indicate the residues experiencing chemical-shift changes by NMR experiments. Numbers along the sequence show residues index.

To examine which computational program is suitable to determine the dynamic network of a protein, we first analyzed the widely studied protein CypA in terms of cross-correlation using conventional molecular dynamics simulations. CypA was once tested as a model protein in which the collective nature of the dynamics and those residues build a common dynamic network via NMR experiments. Among residue mutations in different positions of the dynamic network, R55A localizes in the active site, H70 within loops adjacent to the active site, and K82 is 10 Å distal away from the active site. These mutations have been proven to cause chemical-shift changes in a common network throughout much of the protein (see Figure 1), which is similar to the dynamic networks by relaxation measurements of the wild type CypA. This demonstrates that local perturbations from the mutations involve distal residues that build a dynamic network. Here, we examined the cross-correlated regions regarding the three specific residue mutations in CypA after the cross correlation of the atomic fluctuations of Cα atoms, which was presented graphically in a dynamical cross-correlation map from the last 10 ns equilibrium MD trajectory data to display the correlation of movements of all residues in CypA (see Figure S1). The color-coded stereo structures shown in Figure 2a–c provide a visualization of the correlated motions relative to the three specific residue mutations in CypA, where warm colors (from yellow to red) indicate relatively higher correlation, whereas the cold color (blue) represents highly anticorrelation. Residues that are neither highly correlated nor anticorrelated are colored white. For all the R55A, H70A, and K82A mutations, both highly correlated and highly anticorrelated residues (see Figure 2a–c) were observed. Amongst, highly motion correlated residues relative to R55 (see Figure 2a) include its adjacent residues from F53-I57 (red) to P58-M61 (orange) and a dozen of distal residues E143-S147 (orange), R148-K151 (red), and T152-I156 (orange) whereas S77-Y79, K82-E84 and A103-F113 are anticorrelated with R55 and colored blue, which means opposite directed movements relative to R55, such as the identified correlation network of R55 consisting of highly correlated and anticorrelated regions by MD calculation are largely overlapped with the experimentally dynamic network shown in Figure 1a. The motional correlation regions relative to residue H70 are shown in Figure 2b. Neighboring residues M61-K76 (orange for M61-F66, red for T67-T73 and orange for G74-K76) and a few distal residues T107-A117 (yellow) display positive correlation movements with H70 while the colored-blue residues T152-G162 are anticorrelated with H70. This correlation pattern of H70 is quite similar with the experimental dynamic network of H70 (see Figure 1b). Figure 2c shows that for K82, its neighboring residues G64-H70 (yellow), G75-E84 (orange for G75-Y79 and red for G80-E84), and P105-Q110 (orange for P105-N108 and red for G109-Q110) are positively correlated with K82, but residues F53-P58 (blue) and T152-T157 (blue) have anticorrelated movements relative to K82. The correlation pattern of K82 is quite similar with the experimental dynamic network of K82 (see Figure 1c). Quantification was conducted to evaluate the consistency between the experimental results and the computational results. Figure 2g shows sequence alignment with regions painted in red (representing dynamic networks from cross-correlation analysis) and boxes with diagonal lines (representing dynamic network from the NMR experiments [21]. We can see that the MD simulation identified approximately 85% of residues in the dynamic network detected by NMR experiments, which demonstrates that cross-correlation analysis based on nanosecond timescale MD simulation could mostly reveal the dynamic networks in CypA or proteins with similar feature. Therefore, we observed that the correlated regions of all the three residue mutations in CypA overlap well with the corresponding previous experimentally demonstrated dynamic networks (see Figure 1). This suggests that the dynamic networks can be calculated from the MD simulation trajectories.

### 2.2. The Cooperative Networks in CypA Are also Consistent with the Experimentally Detected Dynamic Networks

To enrich our method to calculate the dynamic network, we also examined the energetically coupled residues relative to a specific perturbation in CypA to see if the cooperative network formed by the energetically coupled residues actually accounts for the dynamic network. A computational algorithm COREX/BEST has been developed to elucidate the cooperative effects in proteins. An ensemble of conformational states was generated using the crystal structure of CypA as a template before the cooperative effects were investigated by substituting one residue and monitoring its effects on all other residues. The effects of a single-site substitution to Ala on all other residues are quantified by calculating the change of free energy.

All three mutations cause mild perturbation on other residues. The free energy changes for R55A mutation range from −0.08 to 0.27 kcal/mol for an H70A mutation range from −0.22 to 0.59 kcal/mol and for a K82A mutation from −0.22 to 0.33 kcal/mol. In this case, we considered the residues with free energy change greater than 0.2 kcal/mol as susceptible residues which are energetically coupled to the mutated residues R55, H70, or K82. The recognized susceptible residues are considered to be thermodynamically coupled with each other and the mutated residue and therefore form cooperative networks.

Figure 2d–f shows the energetic coupling regions (colored in red) detected by the mutations at R55A (Figure 2d), H70A (Figure 2e) and K82A (Figure 2f), respectively. R55A mutation is found being coupled with four regions consisting of residues V20-R37, S51-Q63, S110-T116 and H126-F129. These regions displaying cooperativity will simultaneously respond to a perturbation at a specific site. H70A mutation causes three coupling regions consisting of residues A38-G47, Q63-G75 and I158-G162. In addition, K82A mutation causes two coupling regions consisting of residues G75-E86 and S99-S110. The results suggest that a cooperative network among distal residues can be deduced by single-site substitutions. The COREX/BEST algorithm detected a dynamic network in CypA similar to that by NMR studies (see Figure 1).

### 2.3. The Dynamic Networks in IL-2 Identified from Cross-Correlation Analysis

**Figure 3:**
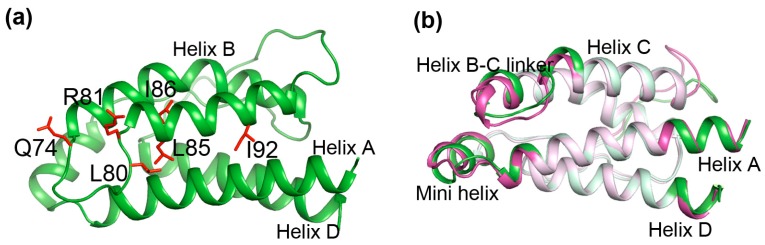
Structural rearrangement resulting from the mutations. (**a**) The crystal structure of interleukin-2 (IL-2). The sidechains of mutated residues are shown as stick type in Red. Q74, L80, R81 are located on the BC loop and L85, I86, I92 are located within the helix C core; (**b**) The structure superposition of IL-2 (green) and D10 (magenta). The completely overlapped regions in the structures are colored in light color, whereas the obvious deviations are highlighted in bright color. The mini helix, the helix B-C linker region and the loop region subsequent to helix C show significant conformational changes. The N-terminus of helix A and the C-terminus of helix D show slight deviations.

The above analysis of CypA by MD and COREX/BEST methods have proven the feasibility of using the two computational approaches to determine the dynamic networks of a protein. We then used both MD and COREX/BEST to detect the dynamic networks in an important type I cytokine interleukin-2 (IL-2). One mutant of IL-2 cytokine named D10 has been developed using yeast display techniques and was termed as a superkine with dramatically enhanced activity relative to wild type IL-2 [12]. Among the six mutated residues in D10 only one residue is located in the binding sites while the other five residues are core region residues and are located at different positions away from the binding sites (see Figure 3a). Investigating the dynamic networks related to the mutations will help us to understand how the mutations resulted in advanced activity. Based on 100 ns all atom MD simulation on an IL-2 molecule, a dynamical cross-correlation map was firstly constructed, which is shown in Figure S1. Overall, both highly correlated (red) and highly anticorrelated (blue) relationships were observed between several separated regions of the protein. From the overall cross-correlation map, highly correlated and anticorrelated motions relative to residues Q74, L80, R81, L85, I86 and I92 were examined carefully and the results are respectively shown in the crystal structure of IL-2.

**Figure 4:**
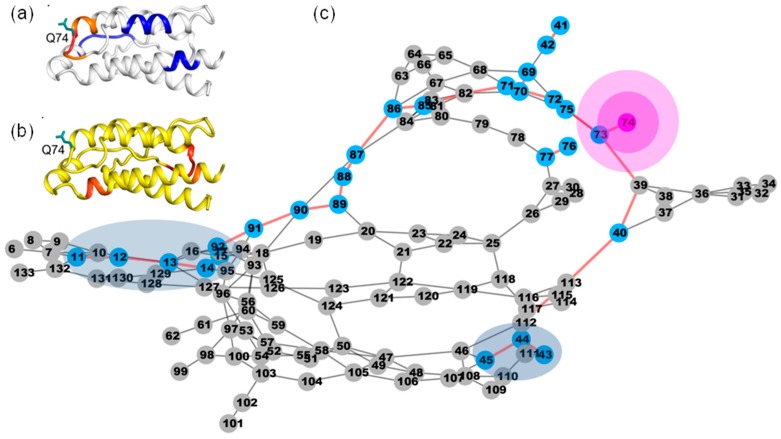
The possible pathway for the propagation of disturbance by residue mutation at Q74 in IL-2. (**a**) The dynamic network in IL-2 deduced by cross-correlation analysis. The dominant correlated pattern of the residues in IL-2 is shown relative to residues Q74. The residues color coded red, orange and yellow are correlated, but these residues are anticorrelated with those color-coded blue; (**b**) The dynamic network in IL-2 deduced by COREX/BEST algorithm. The energetic coupled regions in the network for Q74A are colored in red. For clarity the coupling regions only consist of the residues with the free energy change greater than 1.0 kcal/mol; (**c**) The possible allosteric pathway between the mutation site of Q74 and the coupling regions. Network representation of IL-2. The red bold edges represent allosteric pathways connecting mutation sites (magenta nodes) and coupling residues (blue nodes). The other components are represented as gray nodes.

**Figure 5:**
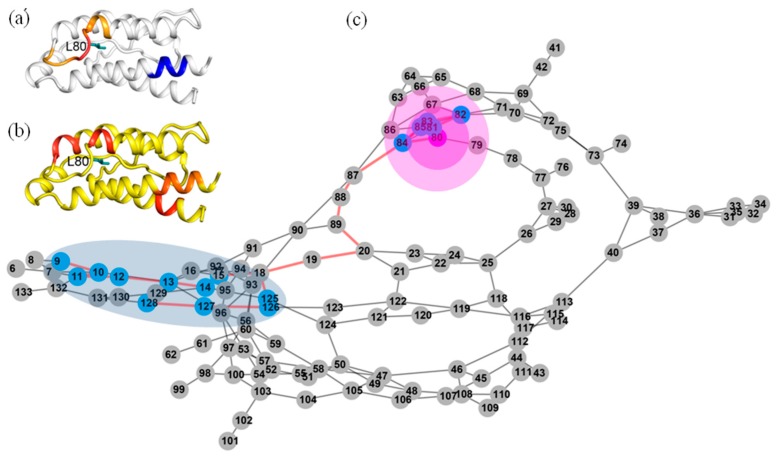
The possible pathway for the propagation of disturbance by residue mutations at L80 in IL-2. (**a**) The dynamic network in IL-2 deduced by cross-correlation analysis. The dominant correlated pattern of the residues in IL-2 is shown relative to residues L80; (**b**) The dynamic network in IL-2 deduced by COREX/BEST algorithm. The energetic coupling regions in the network for L80A are colored in red; (**c**) The possible allosteric pathway between the mutation site of L80 and the coupling regions. The illustration for all graphs is the same as that in Figure 4.

**Figure 6:**
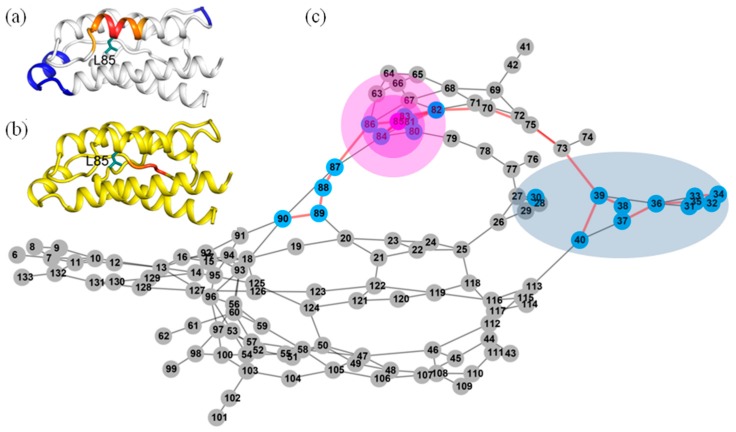
The possible pathway for the propagation of disturbance by residue mutations at L85 in IL-2. (**a**) The dynamic network in IL-2 deduced by cross-correlation analysis. The dominant correlated pattern of the residues in IL-2 is shown relative to residues L85; (**b**) The dynamic network in IL-2 deduced by COREX/BEST algorithm. The energetic coupling regions in the network for L85A are colored in red; (**c**) The possible allosteric pathway between L85 and the coupling regions. The illustration for all graphs is the same as that in Figure 4.

**Figure 7:**
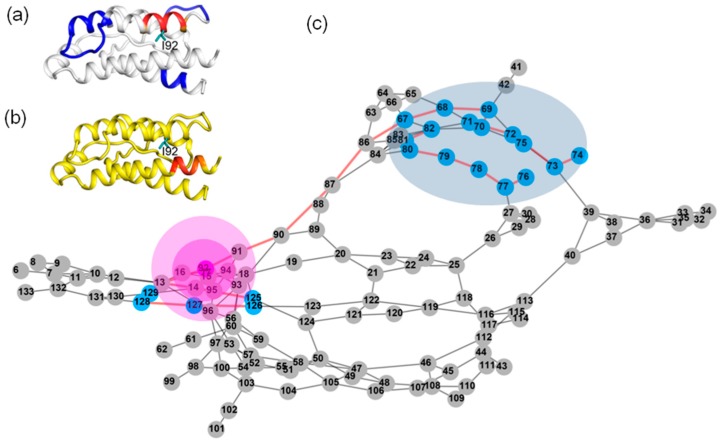
The possible pathway for the propagation of disturbance by residue mutations at I92 in IL-2. (**a**) The dynamic network in IL-2 deduced by cross-correlation analysis. The dominant correlated pattern of the residues in IL-2 is shown relative to residues I92; (**b**) The dynamic network in IL-2 deduced by COREX/BEST algorithm. The energetic coupling regions in the network for I92A are colored in red; (**c**) The possible allosteric pathway between I92 and the coupling regions. The illustration for all graphs is the same as that in Figure 4.

We can see that every residue has quite a few highly anticorrelated residues as well as a few highly correlated neighboring residues. Figure 4a shows the correlation pattern relative to Q74. Its neighboring residues V69-N77 (orange for V69-L72, red for A73-S75 and orange for K76-N77) have positively correlated movement relative to Q74. The blue-colored residues Q11-E15, L40-Y45 and L85-I92 are anticorrelated with Q74. In Figure 5a, the regions are presented according to the motion correlation relative to residue L80. Residues S75-L85 (orange for S75-N77, red for F78-L80 and orange for R81-L85) are positively correlated with L80, but the blue region of residues T10-E15 are anticorrelated with L80. Similar correlation motions relative to residue R81 were observed. The residues S75-L85 (orange for S75-N77, red for F78-L80 and orange for R81-L85) are positively correlated with R81, but the blue-colored residues T10-E15 and N90-E95 have anticorrelated movements with R81. Figure 6a shows the correlation pattern relative to L85. Its neighboring residues L80-N90 (orange for L80-R83, red for D84-I86 and orange for S87-N90) have positively correlated movement relative to L85. The blue colored regions of residues N30-L40 and E100-T102 are anticorrelated with L85. The correlated motion relative to residue I86 was similar to that of L85. Residues E67-L70 (orange) and L80-N90 (orange for L80-R83, red for D84-I86 and orange for S87-N90) are positively correlated with I86, but the blue regions of residues N30-L40 and E100-T102 are anticorrelated with I86. Figure 7a represents the highly correlated and anticorrelated motions relative to residue I92. The residues I89-K97 (orange for I89-N90, red for V91-E95 and orange for L96-K97) are positively correlated with I92, but the blue colored residues E67-P82, E100-C105 and C125-I129 have anticorrelated movements with I92. Those residues that are not involved in the above description are colored white.

**Figure 8:**
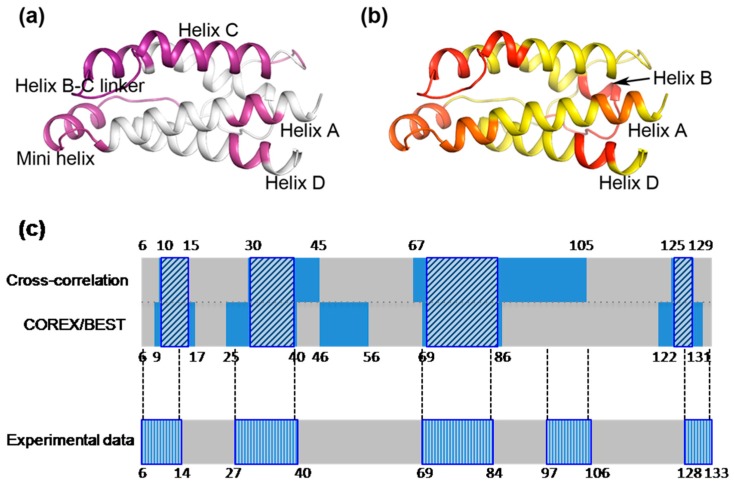
The dynamic network corresponding to the six mutation sites in IL-2 was detected by cross-correlation analysis and COREX/BEST algorithm. (**a**) The motion correlated regions relative to the mutation sites derived from MD simulation are shown in magenta on the cartoon representation of IL-2 structure; (**b**) A combined energetic coupling region based on COREX/BEST algorithm are shown in red on the cartoon representation of IL-2 structure; (**c**) Comparisons of the dynamic networks calculated by cross-correlation analysis and COREX/BEST algorithm, and with the regions undergoing structural changes in the protein crystals. Along the protein sequence, blue regions indicate the residues within the dynamic networks. Boxes with diagonal bars were painted upon the blue regions to indicate overlapped regions of the dynamic networks calculated by these two methods. Boxes with vertical bars indicate the structural change regions from experimental data (Crystal Strucrues). Numbers along the sequence show residues index.

Taking the cross-correlation analysis of the six mutation residues together, we suggest that the regions that are interrelated distribute throughout the entire IL-2 molecule. The identified pairs of regions that are dynamically coupled reveal a dynamic network of covariant regions within the structure of IL-2. In order to specify the overall motional cross-correlation regions caused by the six mutations in D10, we overlaid the cross-correlation maps relative to all the six mutations together and obtained an overall cross correlation map of the mutations (see Figure 8a). This map was compared later with the regions of conformational changes by the mutations.

### 2.4. The Dynamic Networks in IL-2 Deduced According to COREX/BEST Algorithm

In addition, we explored the dynamic networks in IL-2 relative to the site mutations in D10 by COREX/BEST algorithm. According to the current method of the algorithm, not the real residue substitutions in D10 but single-site substitutions to Ala were applied to IL-2 structure before calculating the residue-specific changes in free energy. Therefore, a certain extent of error in the results cannot be avoided. Analysis of these values revealed that the mutations caused different degrees of influence on each residue, which signifies varying tolerance of each residue to the perturbations. In these mutations, the L85A mutation causes slightly weaker effects on all other residues with the change of free energy ranging from −1.01 to 0.87 kcal/mol. The other mutations of Q74A, L80A, R81A, I86A and I92A induce a change of free energy ranging from −1.28 to 1.12 kcal/mol, −1.12 to 1.68 kcal/mol, −0.48 to 1.64 kcal/mol, −2.61 to 2.32 kcal/mol and −0.67 to 1.95 kcal/mol, respectively. For clarity, we chose a cutoff value of 1.0 kcal/mol to highlight the highly affected residues. The residues with absolute change of free energy greater than the cutoff value are considered to be susceptible to the mutations. Figure 4, Figure 5, Figure 6 and Figure 7b shows the structure of IL-2 in which these susceptible residues are colored in red. Each of the mutations resulted in a global effect as a whole. These coupling regions are diffusively distributed throughout the whole structure in a long-range connectivity pattern. R86A mutation induces drastic perturbation in both the intensity and the extent of network. The coupling regions in the cooperative network consist of residues L25-L40, M46-L56 and N71-I86. Since Arg86 is completely buried inside the core of protein, R86A mutation may make a great difference to the interaction of each residue. Strikingly, L85A mutation leads to small effects on all other residues. Only one small coupling region is detected which consists of residues M46-K48 (see Figure 6b). L80A and R81A mutations induce moderate perturbations on all other residues. L80A mutation deduces four coupling regions consisting of residues K9-E15, V69-Q74, P82-L85 and C125-I128 (see Figure 5b). D81A deduces two coupling regions consisting of residues L72-D84 and C125-I129. Both Q74A and I92A mutations induce perturbations in small range of regions. Q74A mutation deduces three coupling regions consisting of residues T10-Q11, N26-N29 and K49-K54 (Figure 4b). I92A mutation only deduces one coupling region consisting of residues K9-L14 (see Figure 7b).

As a direct test of the collective cooperative property of all the related residues IL-2, a combined energetic coupling region including all of the above coupling regions was shown in red on the cartoon representation of IL-2 structure (see Figure 8b). Five coupling regions (comprising residues K9-Q13, L25-T41, M46-L56, V69-I86 and C125-I129) throughout the whole structure collectively represent a dynamic network within IL-2. The results are mostly consistent with that of the above MD simulation study, so that quantification analyses of the dynamic networks from the two different computational methods as well as the regions undergoing structural changes which is experimental were carried out and shown in Figure 8c. The dynamic networks calculated by cross-correlation and COREX/BEST methods colored in blue were first compared with each other, and the consistency between the two is approximately 85 percent. The common regions identified from the two computational methods were, thus, outlined using boxes with diagonal bars. These regions account for approximately 65 percent of the experimental data represented by the boxes with vertical bars. Separately, the regions identified by cross-correlation methods present about 80 percent consistency with the experimental data, while the results from COREX/BEST provide consistency of about 74 percent. The high consistency validates the feasibility of determination dynamic networks using either MD simulation or COREX method, and the existence of the dynamic networks. Both methods identified the same regions of the dynamic network including the N-terminus of helix A and the subsequent mini helix, and the helix B-C linker region, except that the C-terminus of helix C was identified by MD simulation but not by using the COREX/BEST method and the N-terminus of helix B deduced by COREX/BEST but not by MD simulation. The identified common dynamic networks related to the mutated residues in D10 by both methods involve the N-terminus of helix A and its subsequent mini helix region, the N-terminus of helix B, the helix B-C linker region and the C-terminus of helix D.

### 2.5. The Calculated Dynamic Networks in IL-2 Coincide with the Regions Undergoing Structure Alterations Caused by the Mutations

A superkine D10 was screened from the error-prone PCR IL-2 library for its increased binding affinity for a cognate receptor unit IL-2Rβ. It has been suggested that the increased binding affinity of D10 may profit from its structural rearrangement as a consequence of accumulated mutations. D10 of IL-2 mutant has six mutations: Q74H, L80F, R81D, L85V, I86V and I92F. In these mutations, Q74H, L80F, R81D are located on the BC loop and L85V, I86V, I92F within the helix C core (see Figure 3a). We compared the structures of IL-2 and D10 to elucidate the conformational deviations. Shown in Figure 3b is the structural superposition of IL-2 (green) and D10 (magenta). The most overlapped regions of the structures are whitened out, whereas the obvious deviations are highlighted in bright color. Overall, much of the two structures is highly similar. However, there are several small regions where significant deviations occur, including the mini helix, the helix B-C linker region and the loop region subsequent to helix C. Additionally, slight deviations also occur at the N-terminus of helix A and the C-terminus of helix D. We observed that the conformational changes occur not only at the mutation sites but also in the distant regions.

We focused on the locations of structural deviations and the positions covered by the dynamic network. These residues in the dynamic network exhibit consistency of conformational deviations in response to the accumulated mutations. Notably, in all the locations where significant deviations occur, the mini helix and the helix B-C linker region, are located in the dynamic network (comprising residues N26-N33, N71-I86) (see Figure 3b). Even the locations where slight deviations occur, the N-terminus of helix A and the C-terminus of helix D are adjacent to the network (comprising residues T10-L12, Q126-I129) (see Figure 3b). This indicates structural deviations caused by the mutations tend to occur in the dynamic network, which build the correlation between the location of conformational deviations and the position covered by the dynamic network. The perturbations by residue mutations can propagate along the dynamic network, such that the residues in the dynamic network respond with the changes of other residues within the same network even in distal locations. This observation elicited the close correlation between the structure and the dynamics of proteins from a new facet.

### 2.6. Identifying Allosteric Pathways in the Superkine

To reveal how the site mutations transferred long-range allosteric influences and led to dramatic conformational changes in distal regions, we employed graph theoretical methods to present the residue network in IL-2 by treating each residue in protein as a node in the graph, and the connections between pair of nodes as edges. These edge corresponding residue-pairs are weighted according to the pairwise correlation. We adopted the concept of shortest path between nodes in weighted graph to get insights into internal pathways that are important for propagating allosteric signals [34]. The likely allosteric pathway between residues is calculated by the method which shows the propagation through networks of highly correlated neighbors [16,35]. With the graph of residue networks, we calculated and mapped out the potential allosteric pathways that were deemed important for energy propagation from residue mutation sites.

The determined shortest paths were highlighted in red bold lines in Figure 4, Figure 5, Figure 6 and Figure 7c. The magenta node represents the mutation site, the blue nodes represent the coupling regions calculated by the MD cross-correlation analysis (see Figure 4, Figure 5, Figure 6 and Figure 7a) and ensemble algorithm (see Figure 4, Figure 5, Figure 6 and Figure 7b), and the other nodes are in gray. For the residue mutation at Q74, the disturbance by mutation propagates to remote regions along two pathways (see Figure 4c). One is passed from Q74 to distal region along the coupling residues. The other is passed along residues Q74, A73, M39, L40, T113, F117 and F44. Using network analysis of allosteric pathways between IL-2 residues, we find that a group of coupling residues reside directly adjacent to the mutation site L80, and the pathway consisting of residues S87, N88, I89, N29, L19 and L18 is the bridge connecting L80 and the remote coupling regions (see Figure 5c). Allosteric propagation for R81 is similar to that of L80. A group of coupling residues surrounding R81 was found being affected with each other. The same pathway through residues S87, N88, I89, N29, I19 and L18 is found to transfer the effect of R81 mutation to distal regions. Figure 6c shows the highlighted allosteric pathway for L85. The coupling residues surrounding L85 propagate the effect of mutation one by one. Another pathway consisting of residues L70, S75 and A73 transfers the disturbance to remote region. In Figure 3, the disturbance by I86 mutation propagates to distal regions along two pathways. The coupling residues residing adjacent to I86 transfer the effect to the next residue one by one. The effects of mutation at residue I92 (see Figure 7c) propagate along similar pathway to that for Q74, L80 and R81. Residues H16, Q13 and L14 form a potential allosteric pathway connecting residue I92 and the N-terminal segment. In addition, a longer pathway consisting of residue V91, N90, S87 and N88 connects residue I92 and remote regions.

In all, we observed that an allosteric pathway consists of a set of residues in (dynamic) contact in the protein, which act as a strain of energy and radiate out from a mutation site and result in long-range allosteric conformational changes through residue-residue interactions [34].

### 2.7. Most of the Mutated Residues in Superkine are Critical Ones in the Interaction Network

To evaluate the impact of each residue mutated in the superkine, we employed a newly developed graph theory technique and used MD-TASK software to analyze the communication within a protein in terms of betweenness centrality (BC) and the shortest path (L). BC is a measure of how important a residue is for communication within a protein. The BC of a node is equal to the number of shortest paths from all nodes to all others that pass through that node. It provides a measure of the usage frequency of a residue in cross network communication, where high usage residues are considered to potentially play a role in controlling intra-protein communication [30]. Before calculating BC and L, the dynamic residue network needs to be constructed with the normal residue nodes, while only the edge between nodes established if the Cβ (Cβ for glycine) atoms for each residue are within a cut-off distance of 7.0 Å over the simulation trajectory.

**Figure 9:**
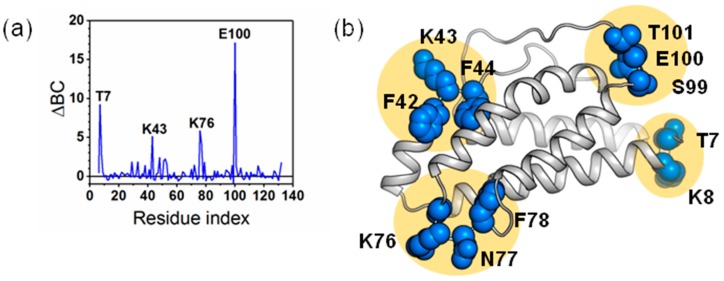
The measurement of betweenness centrality in IL-2. (**a**) Betweenness centrality (BC) profile showing regions in IL-2. Peak locations indicate residues with high frequencies of usage in intra-domain communication; (**b**) Structural mapping of high BC residues. The cartoon presentation of the crystal structure of IL-2 with peak residues shown as blue spheres.

BC is calculated for the protein IL-2 to identify the critical residues for intra-protein communication. The BC profile of IL-2 protein reveals several peak locations (residues T7, K43, K76 and E100) with high BC values (see Figure 9a). These peak residues are mapped to the structure of IL-2 (see Figure 9b). The residues in these regions are identified as critical hubs for the control of intra-protein communication. The mutation sites of Q74, L80 and R81 are located in these regions. Our computational study has implicated these residues in intra-domain communication. BC selects several groups of residues suggesting key intra-domain communication points. These regions correspond to the mutation sites in IL-2 mutant that induced conformational changes.

**Figure 10:**
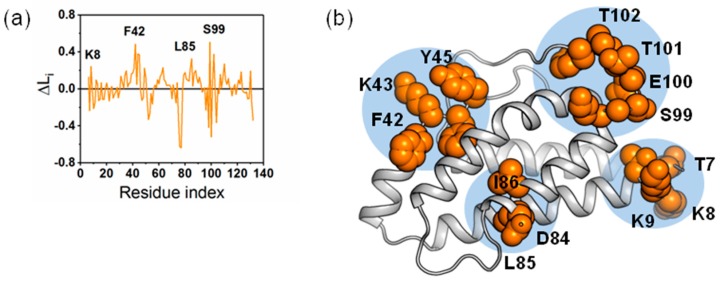
The measurement of reachability in IL-2. (**a**) Change in reachability (ΔL_i_) plots for the protein of IL-2. Regions with high ∆L_i_ are important structural elements for intra-domain communication; (**b**) Structural mapping of high ΔL_i_ residues on the cartoon presentation of the crystal structure of IL-2. The peak residues in (**a**) were shown as orange spheres.

The average shortest path describes how accessible a residue is within the protein. For a given residue, L is the sum of the shortest paths to that residue, divided by the total number of residues minus one. Previous studies have suggested that positions that result in high delta L values may steer conformational changes [30]. The L*_i_* profile for IL-2 protein shows that the protein experiences both positive and negative change. Negative ΔL*_i_* can be described as a shortening of the average path length between all residues and residue i, while positive ΔL*_i_* values indicate increased path lengths. Regions with high L*_i_* are important structural elements for intra-protein communication. The peak locations (K8, F42, L85 and S99) of L*_i_* (see Figure 10a) are consistent with those of BC. These peak residues are mapped to the structure of IL-2 (see Figure 10b). The residues in these regions are implicated in the progression of conformational change. Therefore, we speculated that residues L85 and I86 also contribute to the structural change.

### 2.8. Application of Identifying Distant Structural Effect through Nanosecond Time Scale MD Simulations

Taken the above together, nanosecond MD trajectory for IL-2 is able to efficiently map the dynamics network, and the regions of this network predict the regions that are most likely to undergo structural changes by site mutation. Analysis of critical residues and allosteric pathways based on the MD data in addition to the EAM algorithm validated the efficiency very well. To further demonstrate this notion, we applied similar analysis to IL-1β, which is a similar size of protein with IL-2 but in a folding pattern of β strands.

#### 2.8.1. The Dynamic Network in IL-1β Identified by Cross-Correlation Analysis 

**Figure 11:**
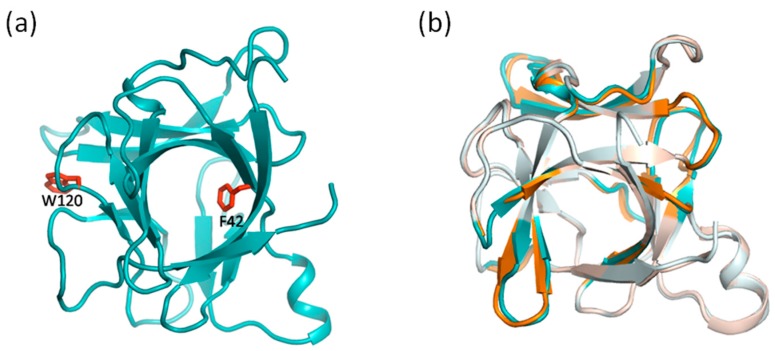
Structural alterations caused by residue mutations in interleukin-1β. (**a**) The crystal structure of interleukin-1β with a solvent-exposed hydrophobic cavity surrounded by β-strands. The corresponding mutated residues in its double mutant (F42W/W120F) are colored in red. The residue F42 is located at a β-strand in the hydrophobic core, and residue W120 locates in a short loop region outside the hydrophobic core; (**b**) Structural superposition of wild-type IL-1β (cyan) and its double mutant (orange). The conformational deviations are highlighted in bright color, while the complete overlapped regions are in pale color. Subtle structural changes occur in some β-strands surrounding the hydrophobic cavity and the short loop regions linking these β-strands.

Interleukin-1β (IL-1β) is a member of interleukin 1 family of cytokines, which plays essential roles in many physiological processes involving cellular activities and inflammatory response. Various mutants by site-specific mutagenesis were obtained to study its structure and function [36]. Since the crucial role of residue F42 in the folding events of IL-1β, a F42W/W120F double mutant of IL-1β was designed to study the effect of the F42W substitution and preserve a single fluorescent probe by W120F at the same time (see Figure 11a).

**Figure 12:**
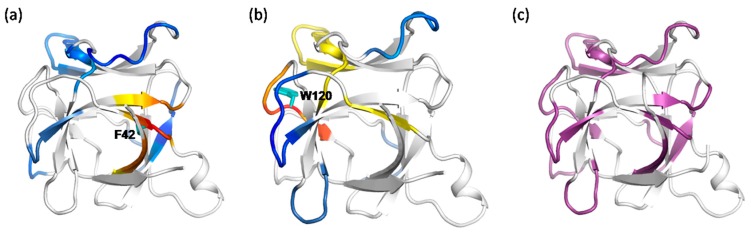
The dynamic network in IL-1β deduced by cross-correlation analysis. (**a**,**b**) The dominant correlated pattern of the residues in IL-1β is shown relative to residues F42 (**a**) and W120 (**b**), respectively. The residues color coded red, orange and yellow are correlated, but these residues are anticorrelated with those color coded blue. (**c**) The motion correlated regions relative to the mutation sites are shown in magenta on the cartoon representation of IL-1β structure, which include all regions mentioned in (**a**,**b**).

Cross-correlation analysis was carried out with the trajectory from molecular dynamics simulations. The dynamical cross-correlation map was obtained to present graphically the cross correlation of the atomic fluctuations of Cα atoms. Correlated motions can be identified in the regions, which move in the same or opposite direction during the simulations. We examined the motion correlation pattern in IL-1β relative to residue F42 and W120 respectively. The color-coded stereo structures shown in Figure 12 provide a visualization of the highly correlated regions, where warm colors (red, orange and yellow) indicate highly positive correlation, cold color (blue) indicates highly anticorrelation, and those residues that show less correlation are colored white. For the residues F42 and W120, both highly correlated and anticorrelated residues were observed. In Figure 12a, the neighboring residues V40-M44 of F42 (orange for V40 and M44, red for V41-S43) and their spatially adjacent residues N7-R11 (orange for N7-L10, yellow for R11) and G61-K63 (orange) are correlated with F42, which reveals that these residues have the same directed movement with F42. The blue regions (L18-G22, N53-P57, C71-T79, K88-K94, K97-V100, E128-M130 and K138-G140) are anticorrelated with F42, which reveals that they have the opposite directed movement with F42. The motion correlations relative to W120 are shown in Figure 12b. The neighboring residues F117-I122 (red for N119-W120, orange for F117-P118 and Y121-I122) as well as residues F42-F46 (yellow), C71-L80 (yellow) and R98-F101 (yellow) are correlated with W120. Residues G49-I56, D86-K92, I106-L110 and A127-M130 are color-coded blue and are anticorrelated with residue W120.

A combined cross-correlation regions shown in Figure 12c includes all regions described above. These cooperative regions are dynamically coupled, which suggests a dynamic network of covariant regions within the structure of IL-1β.

#### 2.8.2. The Dynamic Network in IL-1β Deduced by COREX/BEST Algorithm 

**Figure 13:**
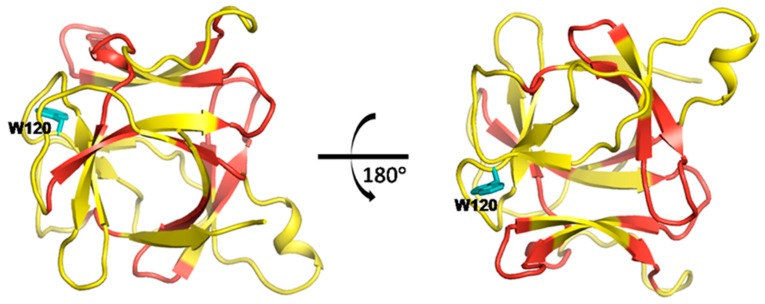
The dynamic network in IL-1β deduced by COREX/BEST algorithm. The identified coupling regions in the networks for W120A are colored in red. The susceptible residues in the coupling regions are identified as the residues with the free energy change greater than 0.6 kcal/mol. The widespread coupling regions suggest that the effects of the mutations can propagate to distal regions, not limited to mutation sites.

Here, we also outlined the dynamic network in IL-1β using COREX/BEST method. We respectively quantified the impact of the F42A and W120A mutations on the rest residues in the protein IL-1β. The free energy changes for F42A mutation range from −0.18 to 0.14 kcal/mol, and for W120A range from −0.80 to 0.61 kcal/mol. Compared to the effect of W120A, F42A mutation almost show no perturbation of any region along the whole structure of IL-1β as indicated by the negligible changes of free energy of the rest residues. This revealed that few residues are susceptible to F42A mutation and no cooperative behavior can be captured in this situation. Thus, we focused on the cooperative information as determined by W120A mutation. We considered the residues with free energy changes greater than 0.6 kcal/mol as susceptible residues which are thermodynamically coupled. These residues in the energetic coupling regions form a cooperative network in the structure of IL-1β. Figure 13 shows the energetic coupling regions (colored red) detected by W120A mutation. The perturbation by mutation caused widespread coupling regions through the whole structure: C8-Q15, L18-K27, V41-M44, D54-V58, L62-Y68, V72-P78, E83-V85, M95-F101 and D142-D145. The result suggested that the effects of the mutations can propagate to distal regions and are not limited to mutation sites. These residues cooperatively behave as a whole in the dynamic network, which collectively respond to the residue mutations.

#### 2.8.3. The Calculated Dynamic Networks in IL-1β Coincide with the Structural Alteration Regions Caused by the Mutations 

A F42W/W120F double mutant of IL-1β was designed to study the effects of key residue mutations on the protein structure. Residue F42 is located at a β-strand in the hydrophobic core, and residue W120 locates in a short loop region outside the hydrophobic core (see Figure 11a). F42 is essential in folding events of protein. W120 is the only fluorescent probe in the wild-type to be used to explore the role of water molecules in stability of IL-1β. The fact that the mutant fold faster than the wild-type implied structural changes that occur in the hydrophobic core.

As the crystal structure of the mutant was determined, we compared the structures of wild-type and mutant. Structural superposition of wild-type (cyan) and mutant (orange) is shown in Figure 11b. The conformational deviations are highlighted in bright color in contrast to the complete overlap in pale color. Structural changes occur in some β-strands surrounding the hydrophobic cavity and the short loop regions adjacent to these β-strands. Compared with the calculated dynamic networks above, the structural changes are observed in the regions where the networks cover. This indicated that the structural alterations caused by residue mutations are likely to occur in the network within IL-1β, which corresponds to the results of computational methods that the impact of residue mutations can propagate to distal regions in a connective pathway.

## 3. Discussion

The structural changes at remote regions (if any) in response to distal residue mutations are realized via certain dynamically cooperative networks. This establishing notion inspired us to unravel the mechanism underpinning allosteric structure alteration of the IL-2 superkine from the dynamic point of view. The alteration renders the mutant structure and became closer to the IL-2Rβ bound state structure of the cytokine and enhanced its binding affinity for IL-2Rβ. As a result, six individual dynamical pathways corresponding to six substituted core residues away from the binding sites to IL-2Rβ were figured out, which formed a dynamic network. The distribution range of the dynamic network coincides with the regions of the structural alteration. Six residues located at different positions resulted in a single dynamically coupling network, and the changes of protein structures mostly fell into the network.

Cross-correlation analysis from MD simulations has long been employed to decipher the dynamic pathways or networks. However, a proper timescale of the MD simulations need to be determined first given that protein motions underlying conformational changes are often on micro- to milli-second timescale. Aided by the previously determined dynamics network related to three-point mutations in CypA, and the structural alterations reflected as a chemical shift changes by the mutations, we proved that nanosecond MD simulations reflect the dynamic networks underlying conformational allostery. For the three single-chain modest-sized proteins consisting of an α-helix (IL-2), β-sheet (CypA, IL-1β) or mixed structures, the dynamic networks derived from either nanosecond MD trajectories or the cooperative network based on EAM remarkably consistent with each other. The regions of the dynamic network are observed to be highly consistent with the regions of the structural alteration. The mutation results in structural organization along the dynamic network pathways. Propagation of structural change in proteins can be described in terms of well-defined dynamic pathways.

Given the fact that residues are connected in sequence or in space, the residues in the coupling regions or correlated subunits build a cooperative network within the protein structure. They can propagate and respond to the disturbance caused by residue mutations. We found that structural changes caused by residue mutations tend to occur in these common regions of dynamic networks. Comprehensive knowledge of the impact of residue mutations on protein structure is essential for gaining a more complete understanding of functions. Apart from experimental mutant structure determination through NMR, X-ray, or cryo-EM measurements, there are a group of programs or Web servers to automate the comparative modeling process, and to evaluate the modeling programs. Although the results are from globular proteins, it might be applied to disordered proteins with further studies. This notion enables us to estimate the possible location of structural reorganization responsive to a certain mutation. The process allows us to understand the mystery of how the residue substitution altered the IL-2 structure from its native state to become similar to the bound state. Explicitly relating the structure alteration with dynamic network could provide clues about which residue would be susceptible to distant residue mutation, and to better understand the special activities of those experimentally obtained protein mutants in the absence of experimental structure.

In addition, the results would potentially enrich our arsenal of rational design. The residues identified as such could reduce the number of mutations that need to be examined and facilitate Alanine scanning. The information in addition to the active site or binding interface can be used in knowledge-based mutagenesis, which is complementary to the more thorough CPD or evolution methods. Computational protein design uses molecular modeling programs to predict an amino acid sequences that will fold into a desired structure. CPD often entails generating protein design candidates by mutating residues on an existing high-resolution structure and then energetically evaluating the designs to find variants that are optimized for certain physicochemical properties such as protein stability or enzymatic activity. For smart response systems design, knowledge-based mutagenesis applies general biochemical principles and knowledge gained from prior studies to guide mutagenesis of native proteins with the goal of achieving improved or novel structural and/or functional properties.

During our preparation of this study, we noticed that discrete molecular dynamics (DMD) simulations with an all-atom force field which originally suits to simulate fast folding of proteins can find out thermal fluctuations heightened between the dynamic coupling residues and the inserted or deleted residues [19]. This coarse-grained approach to determine the networks of residues involved in the transfer of correlated motion across a protein was applied to rescue disease-causative mutant of cystic fibrosis transmembrane regulator (CFTR) [19]. By rationally designing a mutation to this residue, improved aberrant dynamics of mutant CFTR and function of both mutants demonstrate the rescue of a disease mutation by using rational correction of aberrant protein dynamics [19]. Our present result shows a potential integrative approach for the long-range mutant effect prediction, which combines relatively short all atom MD simulations and the coarse-grained algorithm of EAM. The former is adopted to simulate the collective domain motions, while the latter is performed to include (un)folding of the proteins. Through three example systems (IL-2, CypA and IL-1β), we have shown that our combined approach is able to reproduce the structural perturbations of distant mutations as revealed by X-ray structures or NMR chemical shifts by using relative affordable computational approaches to map the dynamics network, which are based on certain distant residues affected by the mutations. Different from Rossetta-Design that the intensive simulation to return the final coordinate of designed protein to users.

This study is only a small step forward in predicting structural alteration from dynamic network, not by new algorithm but validating the potential of the current MD approach. It needs to be specified that only substitution of residues with Ala is available when outlining the cooperative network by COREX methods. This limitation would lead to discrepancy between the calculated network and the real network in the case of IL-2 mutant D10, where each of the six residues was substituted with other residues than Ala. Accordingly, no single cut-off value of free energy change was set to identify the susceptible residues in this case. In this study, we provided a possible explanation of the distal structure changes from the view of dynamic network. Comparing with the available structural coordinate, we found that the structural effects of residue mutations tend to occur in the regions within the dynamic networks, rather than outside the dynamic networks. Our focus is to outline the regions that could be affected by a specific residue mutation, especially the distal regions. At this stage, it is still difficult to predict the exact positions of the atoms after structure alteration caused by the residue mutations merely based on the dynamic networks. Advancing methods to determine the dynamic networks and more details about the relationship between dynamics and structure are warranted before development of practical computational method capable of predicting accurately the changes of protein structures in response to mutations. We expect that similar observations would be much likely obtained with other proteins even though more research for larger proteins is needed, such as determining the appropriate timescale of MD simulation for larger proteins and developing new algorithms o programs to calculate the dynamic networks.

## 4. Materials and Methods 

### 4.1. Structure Preparation

The crystal structures of proteins needed as initial conformations in either cooperative effects or cross-correlation analysis are downloaded from Protein Data Bank: 3K0N for CypA, 1M47 for IL-2, 3QB1 for D10, 1L1B and 1L2H for wild-type and double mutant of IL-1β. The missing loop fragments in IL-2 (S75-K76, S99-T102) and D10 (S99-T102) were modeled using the MODELLER program (MODELLER, University of California San Francisco, San Francisco, CA, USA) [37]. Structural superposition and graphs are performed by PyMOL program (PyMOL, Schrödinger, LLC, New York, NY, USA).

### 4.2. Molecular Dynamics Simulation and the Residue Dynamic Network Therein

#### 4.2.1. Obtaining Trajectories from Nanosecond All-Atomic MD Simulations 

Molecular dynamics simulation was carried out using GROMACS program [38]. The crystal structures of IL-2 and other proteins were used as the initial structural conformations parameterized by AMBER99SB-ILDN force field [39] and placed in a triclinic box and solvated with TIP3P water model [40] with 1.0 nm distance between the solute and box. Corresponding counter-ions were added to neutralize all systems. Energy minimization was performed by the steepest descent algorithm with a tolerance of 1000 kJ/mol/nm and a step size of 0.01 nm. Conformations were equilibrated under an NVT ensemble for duration 300 ps with constant temperature of 310 K and a coupling constant of 0.1 ps, followed by an NPT ensemble for duration 300 ps with constant pressure of 1 bar and a coupling constant of 5 ps. During these two phases, the heavy atoms of the proteins were fixed, which allow the surrounding water to relax with a 2 fs time-step. The van der Waals and electrostatic interactions were identified with cutoff 1.0 nm. The Particle Mesh Ewald (PME) [41] was used for computing the electrostatic interactions in the system with a Fourier spacing of 0.16 nm. The LINCS algorithm [42] was used to constrain all bonds lengths and the SETTLE algorithm [43] was used to constrain the geometry of water molecules. The temperature was controlled by the V-rescale thermostat [44] applied to the protein and non-protein respectively with a time constant of 0.1 ps. And the pressure was controlled by an isotropic Parrinello-Rahman barostat [45] applied to the whole system. Final production MD was performed without position restraints for 100 ns.

#### 4.2.2. Dynamical Network in Cross Correlation from MD Trajectory 

The dynamical cross-correlation map allows the identification of the correlated and anticorrelated motions. To investigate the extent of correlation motions of residues, we analyzed the cross correlation of the atomic fluctuations of Ca atoms reflected in the last 10 ns equilibrium trajectory data, which means that cross-correlation matrices C(i, j) reflecting the fluctuations of the Cα atoms relative to their average position were obtained by the ProDy program (ProDy, University of Pittsburgh, Pittsburgh, PA, USA) [46]. The cross-correlation C(i, j) is given by:

(1) $$C\left(i,\;j\right)=\langle \Delta r_{i}\cdot \Delta r_{j}\rangle /\langle \Delta r_{i}{{}^{2}\rangle }^{1/2}\langle \Delta r_{j}{{}^{2}\rangle }^{1/2}$$

with $\Delta r_{i}$ and $\Delta r_{j}$ representing the fluctuation of atoms i and j from the average position and the angle brackets representing the average over the specific simulation trajectory. Positive C(i, j) values represent a correlated motion between the corresponding residue-pair, while negative values represent an anti-correlated motion. C(i, j) = 1 or −1 means a fully correlated and anti-correlated motion.

### 4.3. Analysis of the Dynamic Network According to Graph Theory but from MD Simulation Data

The overall protein can be treated as a complex graph where individual residues are nodes of the graph and the interactions are modeled as the edges according to graph theory.

#### 4.3.1. Presentation of Dynamic Networks and Determination of Optimal Propagation Paths 

In order to visualize how the perturbation by mutations transfer to remote regions, we turned the interaction network of residues in IL-2 into a graph based on the graph theory and applied the shortest path algorithm built in MATLAB (MATLAB, MathWorks Inc., Natick, MA, USA) to display the nodes and the edges in the networks and optimal paths from the mutation sites to the allosteric regions. Correlation coefficients (C) between the Cα atoms of each residue from the above obtained simulation trajectory was evaluated for the Cα atoms by carefully adjusting the edge weight (E) between two nodes. In more detail, the covariance matrix was constructed according to the formula below:

(2) $$C_{i,j}=\frac{\langle \left(r_{i}-\langle r_{i}\rangle \right)\cdot (r_{j}-\langle r_{j}\rangle )\rangle }{\sqrt{\left(\langle r_{i}^{2}\rangle -\langle r_{i}\rangle {}^{2}\right)\left(\langle r_{j}^{2}\rangle -\langle r_{j}\rangle {}^{2}\right)}}$$

where $r_{i}$ and $r_{j}$ are the cartesian coordinates of atoms i and j, respectively, and bracket-enclosed quantities represent averages over the entire trajectory. We represent the dynamic network as a complete weighted graph. The Cβ atoms in protein represent the nodes and the edges represent the connections between the nodes. The edge weight ($E_{i,j}$) between two nodes is the correlation coefficients ($C_{i,j}$) between the corresponding pair of residues. Since dynamic coupling is mediated by physical interactions between the nodes, we reduced the graph by removing edges between nodes with a contact frequency of less than 0.5 throughout the simulation. We consider two nodes to be in contact if they are within the distance cutoff of 5.7 Å. In graph theory approach, the potential allosteric pathway between residues is described by the shortest path between their respective nodes. To determine optimal paths of perturbation by mutations transfer to remote regions, we applied Dijkstra’s algorithm to the weighted network. The calculation of correlation coefficients and the determination of the shortest pathways are processed by MATLAB.

#### 4.3.2. Recognizing the Critical Residues in the Dynamic Network 

A newly developed program named MD-TASK (MD-TASK, Rhodes University, Grahamstown, South Africa) [30] provides a handy toolset to extract the correlated residues network from MD simulation data and evaluates the network in terms of the changes in betweenness centrality (BC) and average shortest path (L) of each node over the trajectory. BC parameter is used to indicate the number of shortest paths from all nodes to all others that pass through that node. L is calculated by the sum of all the shortest paths to the given node divided by the total number of residues. In this study, we figured out the peak BC and L parameters to specify the most important nodes in the residue interaction network from the above all-atom molecular dynamics trajectory. The Cβ atoms of each residue (Cα for glycine) are treated as nodes within the network, and edges between nodes are established if the nodes are within a distance of 7.0 Å.

### 4.4. Deriving the Dynamics Network from Cooperative Effects Based on Native State Ensembles

To analyze residues that are energetically sensitive to a perturbation by a site mutation form cooperative network or dynamic network within a protein, we employed the COREX/BEST algorithm based on the ensemble theorem to identify the energetic coupled residues by calculating the free energy change of an ensemble caused by the mutations [47], and residues with greater change in free energy are considered to be sensitive to a specific residue mutation.

#### 4.4.1. Generation of the Native State Ensemble 

An ensemble of inter-converting protein structures from the high-resolution crystal structure was built according to COREX/BEST algorithm [48] before the thermodynamic properties of the conformational ensemble were characterized. In more details, eight consecutive residues along the backbone are defined as a “folding unit” (minimum of four residues for the termini considering the end effects) with each folding unit is treated as folded and unfolded states to create all possible conformations. Overall delta G of 5 kcal/mol at simulated temperature of 25 °C were adopted to generate an exhaustive ensemble.

#### 4.4.2. Quantization of Site Mutation Effects on All Other Residues 

To quantify the mutation effect on other residues, single site thermodynamic mutations were first performed before the energy calculation. Briefly, site substitutions to Ala were applied to the native protein structure at each site, one at a time. A residue-level description of the stability of the ensemble, which is known as the residue-specific free energy, was defined as:

(3) $$\Delta G_{f}=-{\mathrm{RT}\text{×}\ln\kappa }_{f,j}$$

where $\kappa_{f,j}$ is the stability constant of residue j. Thermodynamic cooperativity between any residue pair in a protein can be evaluated by the changes in the folding status of residue j upon an energetic perturbation from residue k. The change in free energy at residue j caused by a mutation at residue k can be calculated as:

(4) $$\Delta \Delta G_{j,\mathrm{mutk}}=\Delta G_{j}^{\mathrm{mutk}}-\Delta G_{j}^{\mathrm{WT}}=\left(-\mathrm{RT}\ln\kappa_{f,j}^{\mathrm{mutk}}\right)-\left(-\mathrm{RT}\ln\kappa_{f,j}^{\mathrm{WT}}\right)$$

where $\kappa_{f,j}^{\mathrm{WT}}$ and $\kappa_{f,j}^{\mathrm{mutk}}$ are the stability constants of residues j in wild type protein and a mutant with a mutation at residue k.

## Supplementary Materials

Supplementary materials can be found at http://www.mdpi.com/1422-0067/19/3/916/s1.

## Abbreviations

NMR	Nuclear Magnetic Resonance
CypA	cyclophilin A
IL-2	Interleukin-2
MD	Molecular dynamics
EAM	ensemble allosteric model
