# Supplementary material for: Site-Mutation of Hydrophobic Core Residues Synchronically Poise Super Interleukin 2 for Signaling: Identifying Distant Structural Effects through Affordable Computations
Source: Int J Mol Sci. 2018 Mar 20;19(3):916. doi: 10.3390/ijms19030916 (PMC5877777; doi:10.3390/ijms19030916)
Supplement: Supplementary file 1 [file ijms-19-00916-s001.pdf]

Supplementary Material

Site-Mutation of Hydrophobic Core Residues Synchronically Poise Super Interleukin 2 for Signaling: Identifying Distant Structural Effect through Affordable Computations

Longcan Mei, Yanping Zhou, Lizhe Zhu, Changlin Liu, Zhuo Wu, Fangkui Wang, Gefei Hao, Di Yu, Hong Yuan and Yanfang Cui

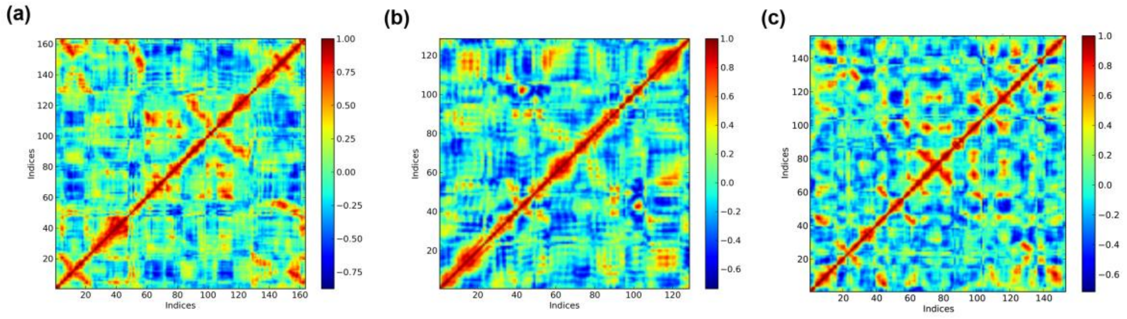

Figure S1. Calculated dynamical cross-correlation maps. The cross-correlation maps of the fluctuation of motion between residues in CypA (a), IL-2 (b) and IL-1β (c), illustrating the correlation of motion between residues in the simulations. The two axes in the maps refer to residues indices. The range of motion is indicated by various colors in the panel. Red indicates positive correlation whereas blue indicates anticorrelation. The coefficients in the legend range from 1 (significantly correlated motion) to -1 (significantly anticorrelated motion). Positive values represent positive correlated motion which indicates residues are generally moving in the same direction, and negative values represent anticorrelated motion which indicates residues are generally moving in opposite directions.
